# Rational Design of Electrode–Electrolyte Interphase and Electrolytes for Rechargeable Proton Batteries

**DOI:** 10.1007/s40820-023-01071-z

**Published:** 2023-04-10

**Authors:** Zhen Su, Haocheng Guo, Chuan Zhao

**Affiliations:** School of Chemistry, Faculty of Science, The University of New South Wales Sydney, Sydney, NSW 2052 Australia

**Keywords:** Energy storage, Proton batteries, Aqueous batteries, Interfacial chemistry, Electrolytes

## Abstract

Rechargeable proton batteries have been regarded as a promising technology for next-generation energy storage devices, due to the smallest size, lightest weight, ultrafast diffusion kinetics and negligible cost of proton as charge carriers. Nevertheless, a proton battery possessing both high energy and power density is yet achieved. In addition, poor cycling stability is another major challenge making the lifespan of proton batteries unsatisfactory. These issues have motivated extensive research into electrode materials. Nonetheless, the design of electrode–electrolyte interphase and electrolytes is underdeveloped for solving the challenges. In this review, we summarize the development of interphase and electrolytes for proton batteries and elaborate on their importance in enhancing the energy density, power density and battery lifespan. The fundamental understanding of interphase is reviewed with respect to the desolvation process, interfacial reaction kinetics, solvent-electrode interactions, and analysis techniques. We categorize the currently used electrolytes according to their physicochemical properties and analyze their electrochemical potential window, solvent (e.g., water) activities, ionic conductivity, thermal stability, and safety. Finally, we offer our views on the challenges and opportunities toward the future research for both interphase and electrolytes for achieving high-performance proton batteries for energy storage.

## Highlights

- The electrode–electrolyte interface reactions (complete desolvation process and incomplete process), interphase design strategies, and advanced interphase analysis techniques for aqueous proton batteries are discussed and reviewed.
- Research progresses on pure phase aqueous electrolytes, hybrid aqueous electrolytes, non-aqueous electrolytes, and solid/quasi-solid electrolytes are summarized.
- Perspectives on both interphase and electrolytes are discussed which can direct researchers to rationally design new interphase and electrolytes for high-performance proton batteries in the future.

## Graphical abstract

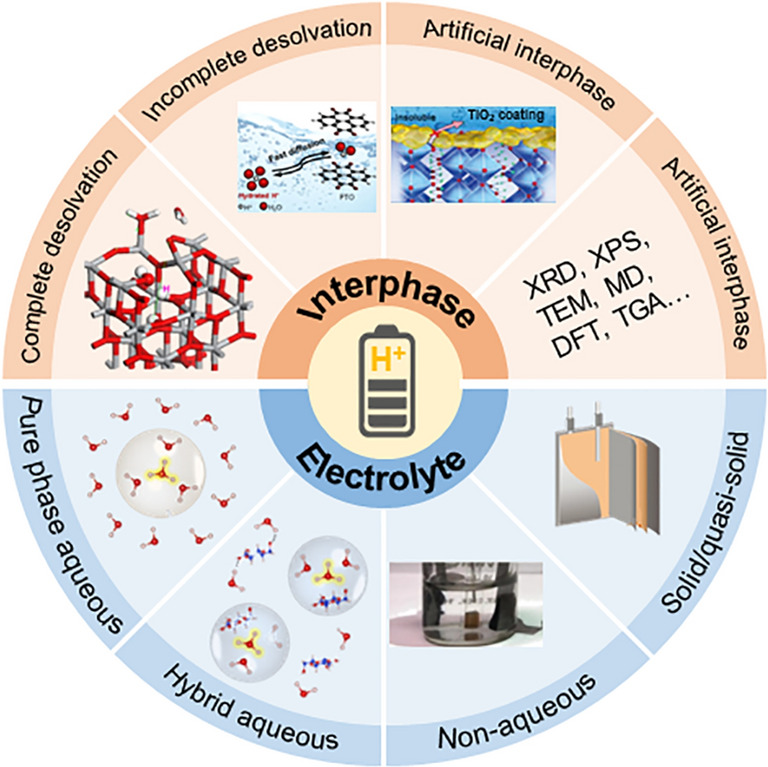

## Introduction

Global warming and shortages of traditional fossil fuels are driving the increasing interest toward renewable energy sources such as solar and wind. The large-scale harvest of intermittent renewables calls for efficient energy storage systems to store, distribute and utilize electricity. Nowadays, rechargeable batteries, especially the lithium-ion batteries (LIBs) have been the dominant energy storage systems [1–3]. However, lithium is not an abundant element on the Earth, and the Li mass fraction in earth crust is estimated to be only 20 ppm [4]. In addition, the inherent safety issue of LIBs associated with non-aqueous electrolytes prevents their application for grid scale storage. The ester-based solvents are highly flammable and reactive with the charged electrodes. The stringent moisture-free process and safety management required for the dangerous and flammable electrolytes also incur substantial costs [5].

**Fig. 1:**
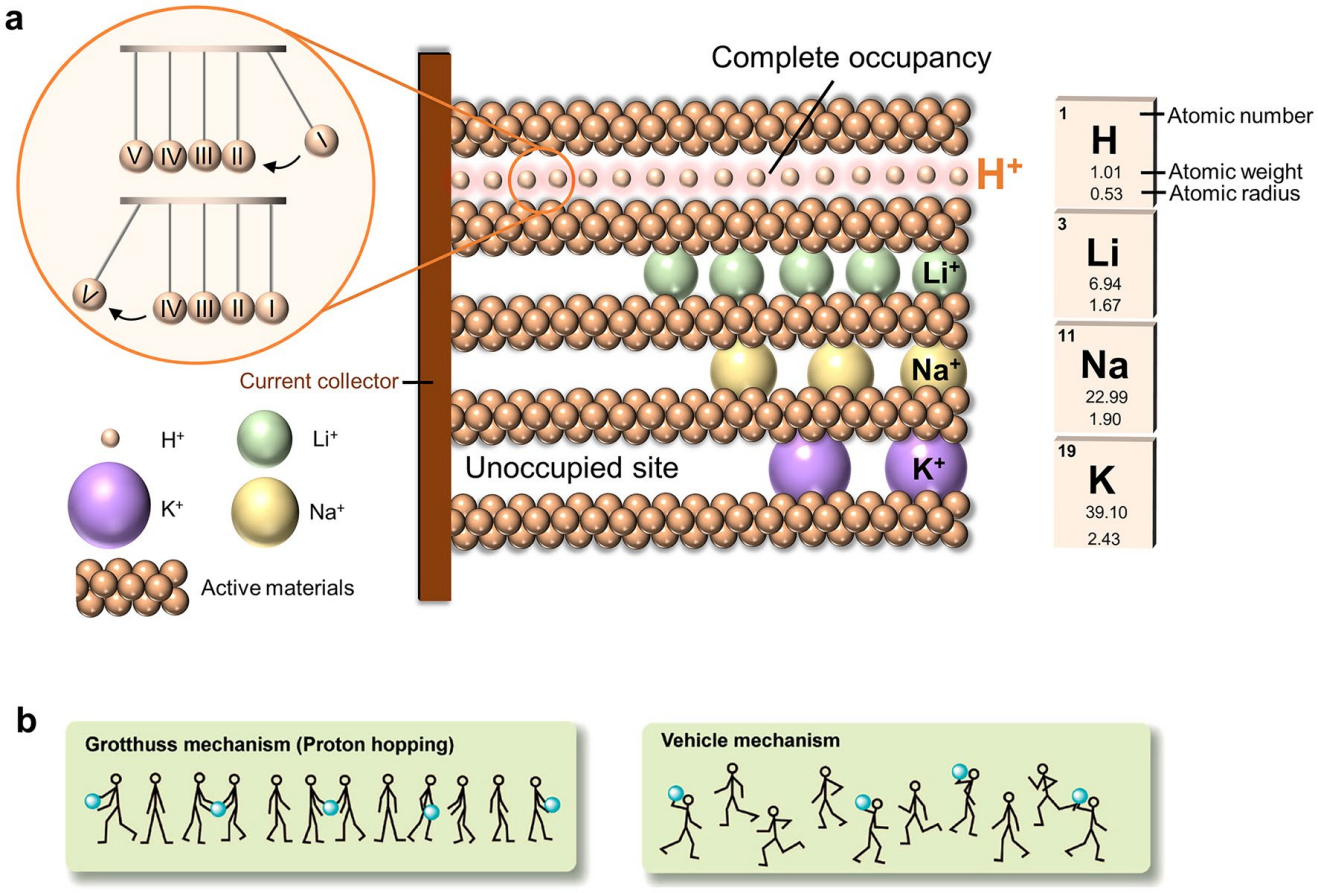
**a** Schematic of the diffusion of different charge carriers in electrodes. Protons with the smallest atomic radius and weight, as well as Grotthuss conduction have great potential to quickly transport in electrodes [21]. Copyright 2020, Wiley–VCH. **b** Illustration of proton conduction in Grotthuss mechanism and vehicle mechanism. The men represent water or base, while the balls represent protons [14]. Copyright 2013, Springer Nature

Aqueous batteries could potentially resolve the above problems, due to the nonflammability, environmental friendliness, and low cost of aqueous electrolytes. Besides, the high ionic conductivity, typically two orders of magnitude higher than organic electrolytes, offers high power densities for aqueous rechargeable batteries. Currently, extensive efforts on aqueous batteries have been devoted to Earth-abundant metallic charge carriers, including Na^+^, K^+^, Zn^2+^, Mg^2+^ and Al^3+^ [6–11]. Although aqueous metal-ion batteries offer high specific capacities, the ion-diffusion kinetics are usually slow because of the increased ionic radius and/or charge number. In this sense, protons (H^+^), which is above lithium on the periodic table, is an ideal charge carrier for aqueous batteries. The advantages of proton batteries include:

i. Protons have the smallest ionic radius and the lowest atomic weight (Fig. 1a). During proton (de)intercalation, the small size not only facilitates a fast diffusion kinetics, but also reduces structure strain leading to a long cycle life. The lightweighted protons can decrease the mass of entire battery systems and induce a higher theoretical capacity when comparing with traditional metallic charge carriers [12].
ii. Protons have the fastest diffusion rate, realized via the unique diffusion-free Grotthuss mechanism, unlike the traditional transport mechanism for metal ions which move in queue [13]. As shown in Fig. 1b, protons hop along the hydrogen-bonded water chain to trigger a series of displacements in the hydrogen-bonding network, similar to the Newton’s cradle (Fig. 1a), leading to a long-range, diffusion-free transport of protons. Protons also can move via the diffusion of loaded polyatomic ions such as hydronium (H_3_O^+^) and ammonium (NH_4_^+^), the so-called vehicle mechanism [14], although the Grotthuss mechanism is much faster than the vehicle mechanism.
iii. Protons can form both quasicovalent hydrogen bonds and ionic bonds with host electrode materials, unlike metal charge carriers with only ionic bonds [12, 15]. The chemical nature of the bonding may induce distinctive reaction thermodynamics and kinetics, bringing new chemistry that is distinct from traditional batteries.
iv. Last but not least, proton batteries have great potential to be used in low-temperature applications, such as in military, polar region, aerospace, and other fields [16], due to the innate low freezing point of acidic electrolytes and fast transport via the diffusion-free conduction in both electrolytes and electrodes. In comparison, most metal-ion rechargeable batteries exhibit poor performance at low temperatures, stemmed from the remarkably decreased electrolyte conductivity and inherent sluggish metal-ion diffusion inside the electrode lattice [17].

Proton battery is a relatively new research area. In 2017, Ji et al. [18] demonstrated the hydronium intercalation into 3,4,9,10-perylenetetracarboxylic dianhydride (PTCDA) in an acidic electrolyte, opening the avenue for promising proton battery research. So far, several material categories have been found showing proton storage capability, including organic solids (e.g., quinones) [18–20], metal oxides (MoO_3_, TiO_2_ and WO_3_) [17, 21–26], two-dimensional transition metal carbides/nitrides (Mxenes) [27], and Prussian blue analogues (PBAs) [28–30]. The recent reported proton-based full cells with their key information are summarized in Table 1. Despite the great progress achieved in the exploitation of electrode materials and electrochemical reaction mechanisms, the reported proton full batteries generally deliver lower energy density (~ 40 Wh kg^−1^) [31] than aqueous lithium-ion (~ 80 Wh kg^−1^) [32] and sodium-ion (51 Wh kg^−1^) batteries [33], not to mention non-aqueous lithium-ion or sodium-ion batteries. In addition, poor cycling stability is another major challenge for most reported proton storage materials. For example, one of the mostly studied electrodes, MoO_3_ can only cycle 100 times in 1.0 M H_2_SO_4_ electrolyte with 67% capacity retention at a rate of 50 C [23]. Recent reviews have comprehensively summarized the progress of proton batteries in electrode materials and their storage mechanisms, including insertion/extraction reaction, chemical conversion reaction and surface redox reaction [12, 16, 34]. Xu et al. [35] reviewed the recent progress of PBAs in aqueous proton battery systems, and summarized the relationship between PBAs structural characteristics and electrochemical behaviors. Liu and coworkers [36] developed an amorphous Al-MoO_x_ anode via a general ion-exchange strategy for proton battery, which delivers remarkable capacity and record-level cycling stability.

**Table 1:** A summary of voltage window, capacity, rate, and cycle life for proton-based full cells

Cathode//Anode	Voltage window (V)	Capacity (based on total mass; mAh g^−1^)	Capacity (based on anode; mAh g^−1^)	Rate (current density; capacity%^a^)	Cycling life (capacity%; cycle number)	References
LiVPO_4_F//MoO_3_	0–1.3	55	n/a	0.093–0.186 A g^−1^; 36%	n/a	[68]
VHCF//MoO_3_	0–1.6	51	n/a	n/a	79%; 1000	[37]
MnO_2_@GF//MoO_3_	0.85–1.55	n/a	190	1–60 A g^−1^; 49%	81%; 300	[17]
H-TBA//MoO_3_	0–1.6	46	n/a	5–100 A g^−1^; 70%	85%; 1000	[22]
MnO_2_@CC//MoO_3_@TiO_2_	0.8–1.6	n/a	200.8	1–20 A g^−1^; 58%	80%; 500	[62]
MnO_2_@GF//TMBQ	0.3–1.3	n/a	320	0.326–32.6 A g^−1^; 46%	77%;4000	[87]
VHCF//PTO	0–1.2	85	270	0.05–37 A g^−1^; 33%	80%; 2000	[44]
MnO_2_@GF//PTO	0.3–1.3	n/a	210	0.16–400 mA cm^−2^; 31%	67%;5000	[19]
MnO_2_@GF//DHAQ	0.2–1.6	n/a	105	0.63–1.13 A g^−1^; 86%	94%;2600	[20]
MnO_2_@CF//ALO	0.4–1.4	n/a	150	5–25 A g^−1^; 81%	63%; 300	[75]
HDC//AC	−0.3–0.7	50	n/a	0.1–1 A g^−1^; 64%	99%; 2000	[60]
PO//PO	0.2–1.2	n/a	147	0.1–2 A g^−1^; 64%	94%; 500	[69]
InHCF//DPPZ	0–1.5	37	n/a	1–10 A g^−1^; 76%	76%; 3000	[48]
ALO//MgMn_2_O_4_	0.1–1.4	n/a	93	0.25–25 A g^−1^; 77%	80%; 30,000	[61]
NiPBA//Ti_3_C_2_T_*x*_ MXene	0–1.2	36	n/a	0.5–5 A g^−1^; 31%	93%; 700	[50]

^a^The capacity retention is calculated by the capacity at the highest current density divided by that at the initial current density. The abbreviations in the table are shown as follows. *PTO* pyrene-4,5,9,10-tetraone, *VHCF* K_0.2_VO_0.6_[Fe(CN)_6_]_0.8_·4.1H_2_O, *GF* graphite felt, H-*TBA* pre-protonated Cu^II^[Fe^III^(CN)_6_]_2/3_·4H_2_O, *CC* carbon cloth, *TMBQ* tetramethylquinone, *DHAQ* 2,6-dihydroxyanthraquinone, *CF* carbon felt, *ALO* alloxazine, *HDC* 2,5-dichloro-1,4-phenylene bis((ethylsulfonyl)amide), *AC* activated carbon, *PO* poly(2,9-dihydroquinoxalino[2,3-b]phenazine), *InHCF* indium hexacyanoferrate, *DPPZ* dipyridophenazine, *NiPBA* PBA-like nickel Turnbull's blue analog

Besides electrode materials, the importance of non-electrode components of proton batteries, such as the design of electrode–electrolyte interphase and electrolytes, has been noticed recently and unveiled gradually. At the electrode interphase, the interactions between solvent molecules and electrode surface can strongly affect the structure stability of surface lattice and subsequently the whole battery lifespan [37]. Meanwhile, interfacial reactions control the charge/discharge rate performance and can be the rate-limiting step in some battery systems [38–40]. Electrolytes play a critical role in proton batteries. Rationally designed electrolytes can suppress the solvent-electrode interactions and protect the electrode structure [24, 41]. In addition, the electrochemical potential window of an electrolyte has a remarkably impact on the energy output by having a high cell potential. In this review, we systematically summarize the progress in the design of interphase and electrolytes for proton batteries. We focus on rechargeable “rocking-chair” proton batteries with only protons/hydroniums as charge carriers, not involving co-insertion of other metal ions. We discuss the desolvation process, solvent-electrode interactions, interfacial reaction kinetics, artificial electrode interphase, and analysis techniques for the electrode interphase. Four major classes of electrolytes (namely pure acid, hybrid aqueous, non-aqueous, and solid/quasi-solid electrolytes) are categorized and reviewed. Perspectives for future design of interphase and electrolytes are discussed for achieving high performance proton batteries and energy storage.

## Electrode–Electrolyte Interphase

**Fig. 2:**
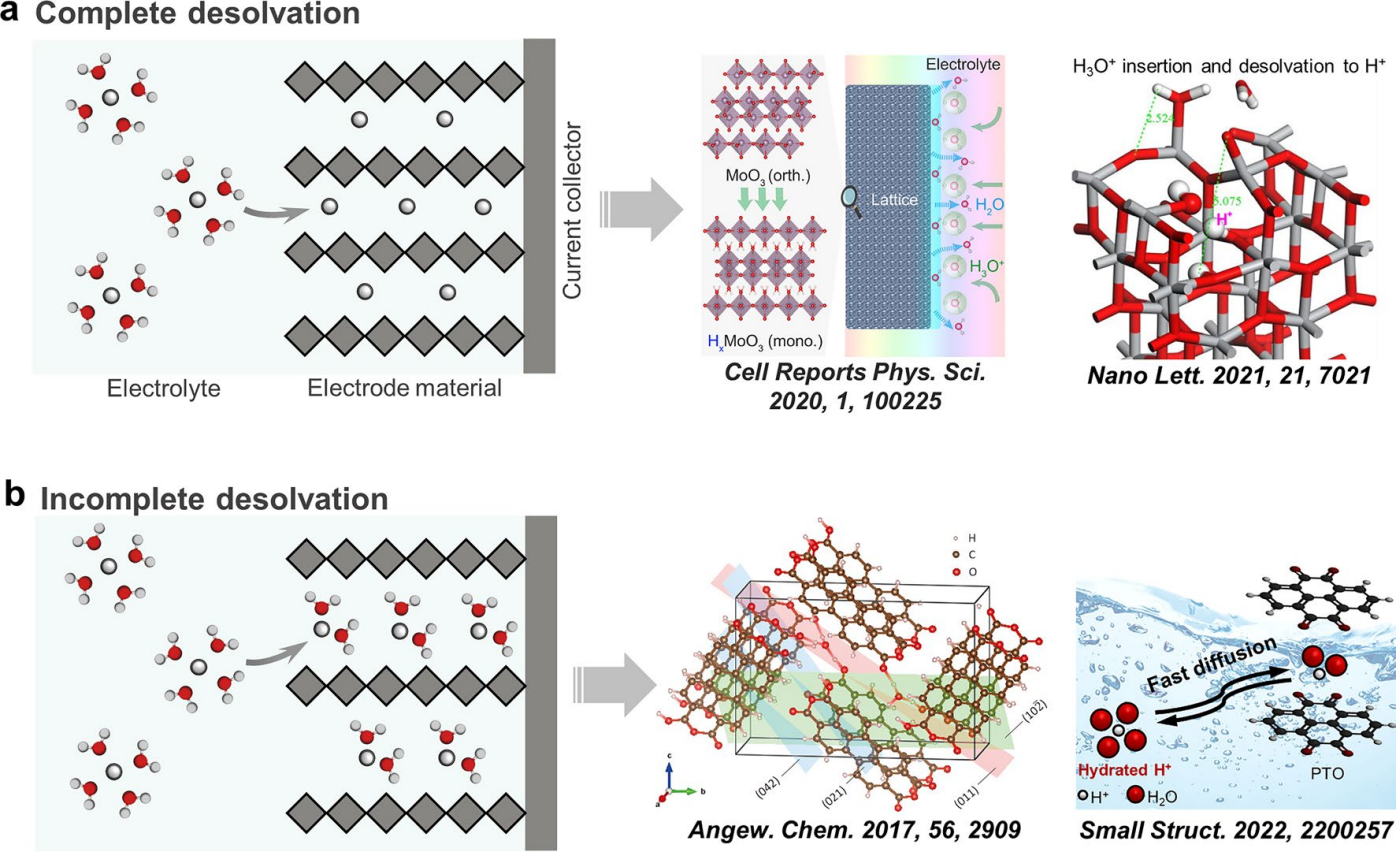
Illustration of two types of desolvation process at the electrode–electrolyte interface: **a** complete desolvation and **b** incomplete desolvation process

H_2_SO_4_ is the most commonly used electrolyte in proton batteries studies. In H_2_SO_4_ electrolytes, the charge carrier exists in the form of hydronium ions (H[H_2_O]_x_^+^), rather naked protons (H^+^) because of the high dehydration energy of hydronium ions (e.g., 11.66 eV for H_3_O^+^ [42]). In 4.2 M H_2_SO_4_, the H_3_O^+^ remains well-hydrated with three H_2_O molecules in the solvation sheath revealed by classical molecular dynamics simulations [43]. During the charge/discharge process, at the electrode–electrolyte interface, the water-solvated protons typically undergo a desolvation process before inserting into the electrode bulk lattice [38]. We summarize the interfacial interactions into two reaction mechanisms: complete desolvation process and incomplete desolvation process. The complete desolvation and intercalation of naked protons, such as that reported for orthorhombic MoO_3_ (*α*-MoO_3_) and anatase TiO_2_ [24, 26], are energetically and kinetically far more difficult and usually leads to a high interfacial resistance (Fig. 2a). The co-insertion of H_2_O with protons (hydronium ions), namely the incomplete desolvation, can significantly reduce the desolvation enthalpy and the interfacial resistance (Fig. 2b). This mechanism has been observed for WO_3_·0.6H_2_O and organic solids [18, 25, 44].

### Complete Desolvation Process

*α*-MoO_3_ has been extensively investigated as an anode material for rechargeable proton batteries because of its stable layered structure in acidic environment, high specific capacity (~ 210 mAh g^−1^), and relatively low reaction potential (−0.5–0.3 V vs. Ag/AgCl). However, it suffers from poor cycling stability [5, 17, 22–24]. In H_2_SO_4_ electrolytes, water molecules from electrolyte and those after desolvation attack the electrode surface together, leading to structural destruction in surface lattice and the slow material dissolutions [24]. Electrochemical quartz crystal microbalance (EQCM) measurements reveal water adsorption on electrodes at the first cycle followed by water adsorption/desorption during following proton (de)intercalation process [24]. Thus, only ~ 20% of capacity remains after 200 cycles in the 1.0 M H_2_SO_4_ electrolyte. The naked protons intercalate into *α*-MoO_3_ lattice, causing the phase transitions between *α*-MoO_3_ and corresponding hydrogen molybdenum bronzes (e.g., H_0.31_MoO_3_, H_0.95_MoO_3_, H_1.68_MoO_3_). It is worth noting here the insertion of naked protons, rather hydronium ions, into the bulk *α*-MoO_3_ lattices suggests complete desolvation of hydroniums.

When using the highly concentrated phosphoric acid as the electrolyte (9.5 M H_3_PO_4_), the interface exhibits some differences [5]. The 9.5 M H_3_PO_4_ electrolyte shows faster interfacial kinetics than 1 M H_3_PO_4_, where protons are solvated by water molecules forming a tetrahedron H_9_O_4_^+^ ions. Protons in 9.5 M H_3_PO_4_ (water-in-acid) resemble the scenario of water-in-salt electrolyte [5] and have incomplete solvation shells with phosphate ions inside and less water molecules in the shell. The direct interactions between the proton and surface oxygen atoms of *α*-MoO_3_ allow direct diffusion from O in H_3_PO_4_ to *α*-MoO_3_ via the hydrogen bonding, leading to the fast charge transfer through the inner Helmholtz layer [5]. Thus, 9.5 M H_3_PO_4_ displays an extremely low charge-transfer resistance of 4.5 Ω in contrast to 10.8 Ω in 1 m H_3_PO_4_, and a high rate performance up to 500 C (100 A g^−1^; 140 mAh g^−1^) with the discharge completed in 5.4 s.

Anatase TiO_2_ also has attracted attentions for electrochemical proton storage because of its lower redox potential than *α*-MoO_3_ (−1.15 to −0.2 V vs. Ag/AgCl), low activity for hydrogen evolution reaction (HER), and low cost. Geng et al. [26] investigated anatase TiO_2_ as a anode and revealed complete desolvation of hydronium ions to enable naked H^+^ insertion. Ex situ X-ray diffraction (XRD) and transmission electron microscope (TEM) show strain-free characteristics of anatase TiO_2_ with no phase transformation, negligible volume change, and invariable interplanar spacing during the charge/discharge process. This suggests the intercalation of naked protons inside bulk lattices because large size hydroniums (e.g., ~ 1.0 Å for H_3_O^+^, close to that of naked Na^+^) would result in clear volume change. Density functional theory (DFT) calculations using H_7_O_3_^+^ as the hydronium model reveal the surface desolvation of hydroniums is dependent on the facets of anatase TiO_2_. The highly reactive TiO_2_ (001) surface impels the desolvation of H_7_O_3_^+^ into H^+^, H, and OH (Fig. 2a). The H and H^+^ bond to the surface oxygen atom of anatase TiO_2_ and the OH bonds to the titanium atom, then naked H^+^ intercalates into the anatase TiO_2_ lattice [26].

Prussian blue analogues (PBAs) represent a group of metal hexacyanoferrates and their general formula is expressed as A_*x*_M[Fe(CN)_6_]_*y*_·*z*H_2_O, where A represents alkali ions and M represents transition metal ions [45–47]. Because of the appealing 3D open channels, PBAs have attracted a lot of attention as promising battery electrodes [28, 45]. A salient advantage of PBAs for proton batteries is the structural resistance to corrosive acids, owing to their strong Fe-CN coordination bonds and exceptionally low solubility product constant (*K*_*sp*_). To date, the reported PBAs for proton storage include Cu[Fe(CN)_6_]_0.63_·□_0.37_·3.4H_2_O (□ represents a ferricyanide vacancy), Ni[Fe(CN)_6_]_2/3_·4H_2_O, K_0.2_VO_0.6_[Fe(CN)_6_]_0.8_·4.1H_2_O, Mn[Fe(CN)_6_]_0.63_·□_0.37_·3.4H_2_O, Zn_3_[Fe(CN)_6_]_2_, Na_2_Mn[Fe(CN)_6_]·2H_2_O, and In[Fe(CN)_6_] [28–30, 41, 48, 49]. The inserted ions is generally attributed to naked protons through Grotthuss conduction in host materials, although their interfacial reactions are unexplored [50, 51]. In weak acid electrolytes (e.g., acetic acid), Gavriel and coworkers [50] have discovered the co-insertion of protons and hydronium ions into the zeolitic sites of Ni[Fe(CN)_6_]_2/3_·4H_2_O via a vehicle-type mechanism. These two insertion pathways (H^+^ by Grotthuss conduction; H_3_O^+^ by vehicle conduction) contribute more charge storage and higher capacity for Ni[Fe(CN)_6_]_2/3_·4H_2_O when using acetic acids.

### Incomplete Desolvation Process

Tungsten oxide and its hydrates (WO_3_·*x*H_2_O, 0 ≤ *x* ≤ 2) have been recognized as promising electrodes for proton storage due to their versatile crystal structures, tuneable water content, and high cycling stability [12]. The hexagonal WO_3_·0.6H_2_O has a tunnel structure with the zeolitic water existing along the dodecagon tunnels [25]. Jiang and coworkers revealed a dynamic water migration process during cycling by the EQCM and ex situ XRD [25]. During the discharge process, the electrode undergoes a three-stage protonation process: (*i*) proton insertion accompanied with the crystal water expelling; (*ii*) naked proton insertion; (*iii*) hydronium (H_3_O^+^) insertion. The WO_3_·0.6H_2_O structure first contracts along the *c*-axis corresponding to the water expelling and later expands along the *ab* planes associated with water ingression. This water migration into/out the electrode lattice suggests the incomplete desolvation process at the electrode–electrolyte interface.

Organic solids are also suitable for storing protons as their relatively spacious interstitial sites, tuneable reduction potential, synthetic availability, low cost, and lightweight [52–54]. Crystalline PTCDA anode has been demonstrated for reversible electrochemical hydronium ion storage [18], hinting the incomplete desolvation of protons at the interface. Upon the intercalation of hydronium ions, PTCDA undergoes significant structure dilation as revealed by the ex situ XRD. As shown in the simulated cell (Fig. 2b), hydronium ions remain in the interstitial space along the (011) planes between stacked PTCDA molecules and are supported by nearby carbonyl groups. Quinones, a common motif comprised of the 1,2-benzoquinone or 1,4-benzoquinone units [55–58], store protons by a typical “proton-coordination” mechanism, where protons coordinate to the negatively charged oxygen atoms of carbonyl groups upon electrochemical reduction, and uncoordinate reversibly upon the electrochemical oxidation.

Pyrene-4,5,9,10-tetraone (PTO) exhibits remarkably high theoretical specific capacity (409 mAh g^−1^), relatively low reduction potential (0.5 V vs. standard hydrogen electrode), and less solubility [59]. Our group has reported the co-insertion of water molecules and proton into the PTO anode and revealed the incomplete desolvation process and effectively reduces interfacial resistance (Fig. 2b) [44]. An outstanding rate performance up to 250 C and as short as 7 s per charge/discharge is achieved. Besides carbonyl-based organic materials, 2,5-dichloro-1,4-phenylene bis((ethylsulfonyl)amide) and alloxazine (ALO) based on active nitrogen center also have been developed for hydronium ion storage [60, 61].

### Artificial Electrode Interphase

**Fig. 3:**
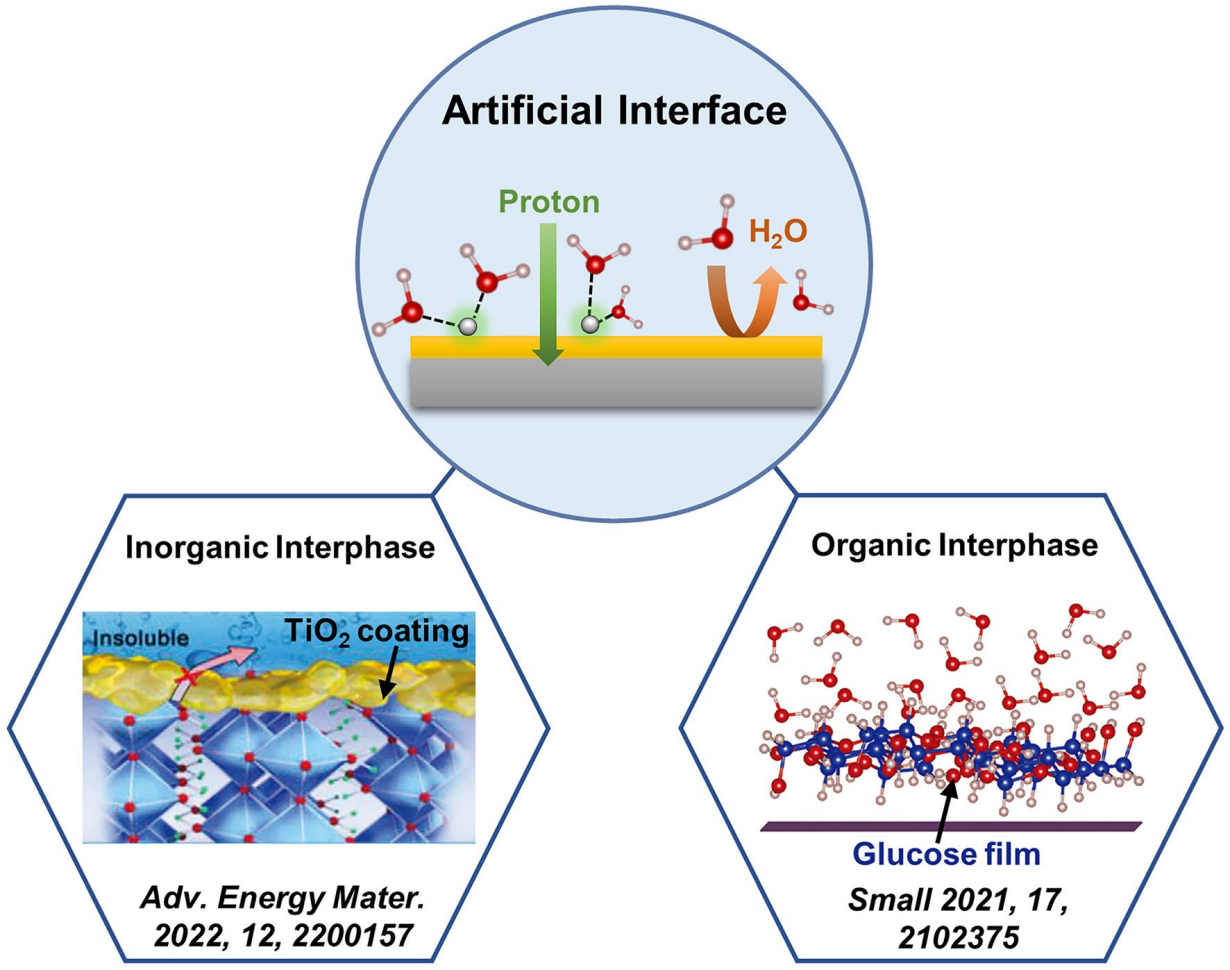
Schematic diagram of the design of electrode interphase to improve electrode stability and interface reaction kinetics

The investigations on the electrode–electrolyte interface for rechargeable proton batteries and the strategies for the design of electrode interphase have been limited. *α*-MoO_3_ is a promising proton battery anode but suffers from the severe material dissolution upon cycling in the default H_2_SO_4_ acid, due to the continuous attack of water molecules from both electrolyte and proton solvation shell on the *α*-MoO_3_ surface lattice (Fig. 3) [24]. Another bottleneck for *α*-MoO_3_ is the large interfacial energy barrier needed for complete desolvation of hydrated protons [62]. Therefore, rational design of electrode interphase can not only protect the electrode surface lattice, but also reduce the interface resistance leading to improved battery cycling lifespan and rate capability. As schematically presented in Fig. 3, the current artificial electrode interphase includes the inorganic layer (e.g., TiO_2_) and the organic layer (e.g., glucose).

Wang and co-authors have coated an ultrathin TiO_2_ shell on *α*-MoO_3_ nanorods to suppress the detrimental dissolution of *α*-MoO_3_ and facilitate the desolvation process of hydronium ions [63]. The TiO_2_ coating is uniform, ultrathin (~ 5 nm), and amorphous, which was fabricated by the coating of the hydrolysis product of tetrabutyl titanate and the later calcination in air. Such TiO_2_ layer protects *α*-MoO_3_ surface lattice and inhibits the dissolution of *α*-MoO_3_ during cycles. MoO_3_@TiO_2_ is found to deliver a high cycling stability of ~ 78.2% capacity maintained after 2000 cycles compared with the case of *α*-MoO_3_ (only ~ 4.5%). The apparent activation energy for the desolvation process on MoO_3_@TiO_2_ is lower than that on *α*-MoO_3_ (4.1 vs. 5.9 kJ mol^−1^). DFT calculations also reveals the smaller desolvation energy of TiO_2_ (~ 1.5 eV) compared with the H_x_MoO_3_ (~ 2.2 eV). The fast desolvation process and superior interphase reaction kinetics plus the good electron/ion conductivity of TiO_2_, enable the MoO_3_@TiO_2_ composite outstanding rate performance (171.0 mAh g^−1^ at 30 A g^−1^). Constructed with MnO_2_, the full cell holds a high energy density up to 252.9 Wh kg^−1^ and power density of 18.3 kW kg^−1^ [63].

Apart from the above electrode material design, electrolyte engineering is another direction to tune the artificial interphase. Our group [43] has developed a “water-in-sugar” electrolyte with high concentration of sugar (e.g., glucose) dissolved in aqueous acids (e.g., H_2_SO_4_, H_3_PO_4_, and HCl). During the charge/discharge process, glucose in the electrolyte binds preferentially to the *α*-MoO_3_ electrode surface. An amorphous glucose-derived organic thin film (~ 5 nm) is electrodeposited on electrode surface, which can block the direct water interactions with the electrode surface. Together with the decreased free water fraction in the electrolyte, water interactions with the electrode surface can be significantly suppressed. The electrode surface lattice is stabilized with higher structural order and less *α*-MoO_3_ dissolution, thus realizing the remarkably enhanced cycling performance for proton storage (over 100,000 times) [43]. Similarly, other electrolytes have been attempted to form an organic interphase for proton batteries, including hydrogen-bond disrupting organic molecules glycerol, methanol, ethanol, acetic acid, and ethylene glycol, which also prefer to bind with *α*-MoO_3_ surface and protect it from water attacking [37].

### Interphase Analysis Techniques

**Fig. 4:**
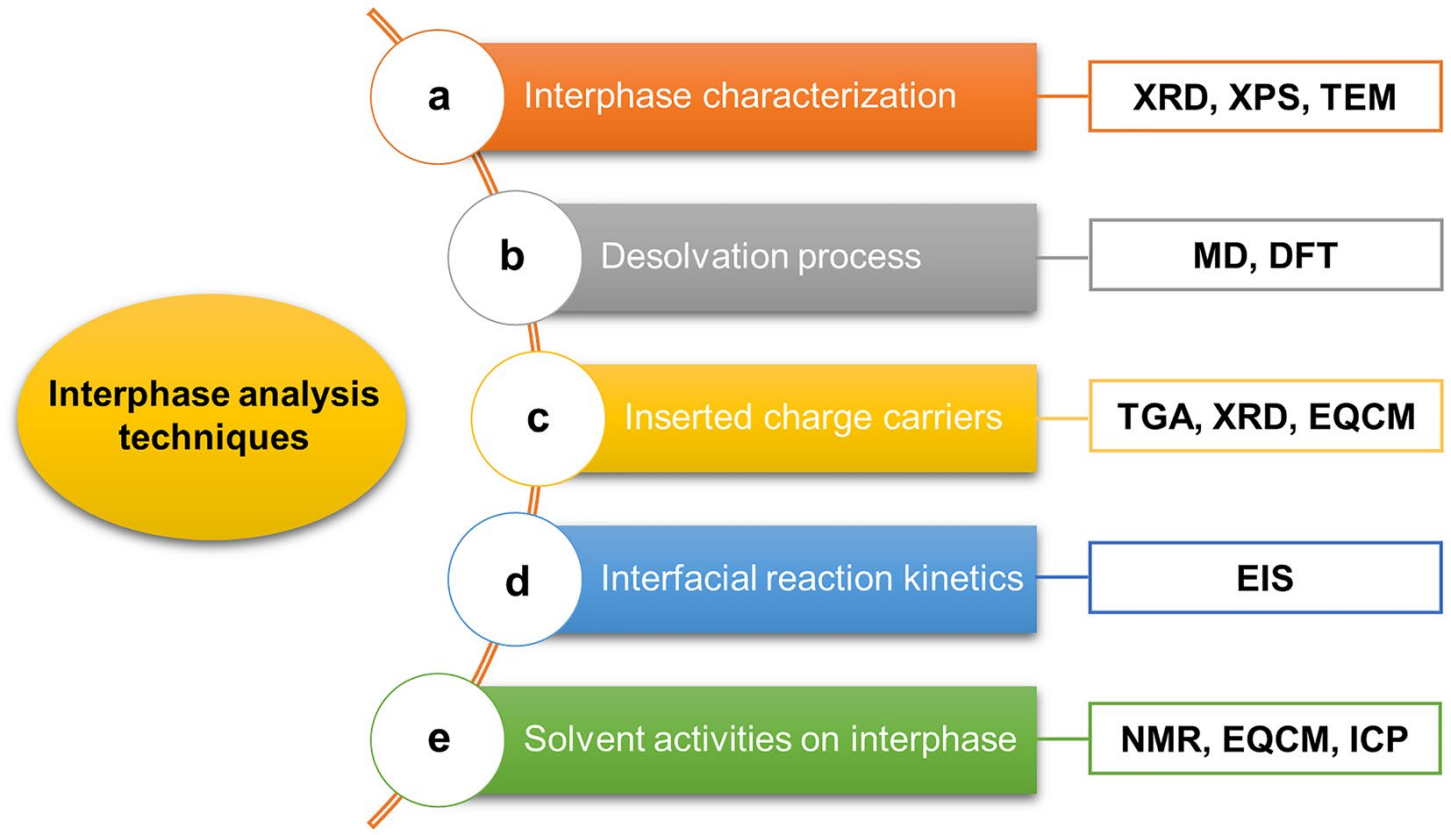
Characterization techniques, electrochemical measurements, and computational simulations for the analysis of electrolyte–electrode interphase

Given the field of proton batteries is still in its infancy, a review of the analytical techniques would provide guidelines for the accurate evaluation and analysis of proton battery interphase, and also spur the development of high-effective interphase in the future. To qualitatively or quantitatively analyze the electrode–electrolyte interphase and to evaluate the effectiveness of interphase modification approaches, different characterization techniques and electrochemical measurements as well as computational tools have been applied to the electrode interphase. We summarize five categories of analysis techniques for the study of the basic interphase characterization, desolvation process, interfacial reaction kinetics, solvent activities on electrode surface, and identification of inserted charge carriers (Fig. 4).

i. For the phase characterization of interphase, commonly used characterization techniques include XRD, X-ray photoelectron spectroscopy (XPS), and TEM with energy dispersive spectroscopy (EDS) mapping. The phase and crystallinity of the interphase could be studied by XRD and TEM techniques. TEM with EDS mapping images can reveal the morphology, thickness, and element content of the interphase. XPS can also confirm elements and valence states of interphase components.
ii. The desolvation process at the interphase is difficult to be directly uncovered by experimental techniques. Computational simulations such as molecular dynamics (MD) simulations and DFT calculations can help to understand the interface reactions. MD simulations have been conducted to simulate the solvation structure of protons in electrolyte systems. DFT calculations have been applied to investigate the detailed desolvation process and hydronium ion diffusion pathways at the interphase, such as the hydronium ion desolvation energy and intercalation energy barriers. Through DFT calculations, Geng et al. revealed that hydroniums first desolvate into H^+^, H, and OH at the anatase TiO_2_(001) surface, followed by the H^+^ intercalated into bulk lattice [26].
iii. Identification of inserted charge carriers (naked protons or hydronium ions) into electrode lattices can reflect the desolvation process to be complete desolvation or incomplete desolvation. Characterization techniques including thermogravimetric analysis (TGA), XRD, and EQCM can be applied. For TGA spectra, the weight loss in the range of 120–280 °C can be ascribed to lattice water by comparing the data of both initial and cycled electrodes. Ex situ or in situ XRD can monitor the phase transformation and structure evolution of electrode materials [12, 18, 24], where the large volume expansion typically suggests the large-sized hydronium ion insertion while no phase transformation and negligible volume change normally indicates naked proton intercalation. In addition, EQCM is a powerful technique to offer valuable insights on charge carriers, which can serve as an in situ gravimetric probe for electrodes during cyclic voltammetry (CV) tests [12].
iv. Electrochemical impedance spectroscopy (EIS) is a powerful technology to study the interfacial reaction kinetics, where charge-transfer resistance (*R*_*ct*_) can be obtained by the small diameter of semicircles at the high-medium frequency region of EIS spectra and reflects the interface resistance [37]. *R*_*ct*_ at different temperatures can also be obtained to calculate the apparent activation energy for the desolvation process to investigate the desolvation kinetics of hydrated protons [63].
v. For solvent activities on electrode surface, the commonly used characterization techniques are ^1^H solid-state nuclear magnetic resonance (NMR), EQCM, inductively coupled plasma-optical emission spectroscopy (ICP), Raman, XRD, and SEM. For example, *α*-MoO_3_ suffers from water attack on surface lattice and the accompanying material dissolution. ^1^H solid NMR of cycled electrodes can directly distinguish the H signal from the surface adsorbed water species on *α*-MoO_3_ surface [24, 37]. The specific water content involved on electrode surface can be monitored by EQCM through the mass change of electrodes [24, 43]. Other characterization techniques (including ICP, Raman, XRD, and SEM) can reveal the electrode material dissolution, surface structure distortion and morphology damage of electrode surface caused by the solvent interactions.

## Electrolytes

**Fig. 5:**
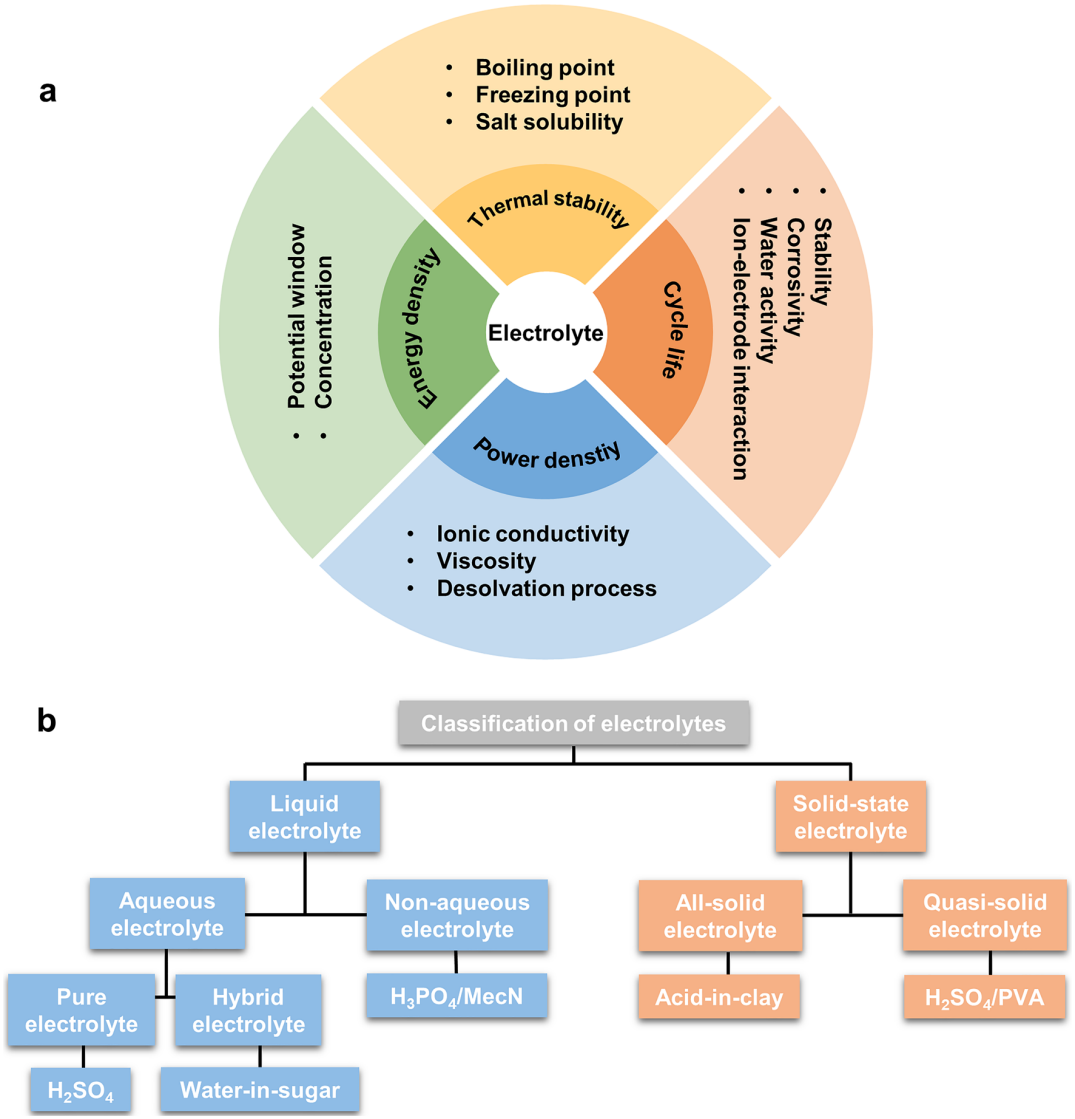
**a** Physicochemical and electrochemical effects of electrolytes on proton batteries. **b** Classification of electrolytes for proton batteries

Electrolytes play a critical role in proton batteries, which not only conduct charges between cathodes and anodes, but also determine other important properties for battery systems, such as the thermal stability, internal resistance, power density, energy density, and cycle life (Fig. 5a) [5]. Freezing point and boiling point of an electrolyte significantly affect the thermal stability of the battery and determine its operation temperature. A high conductive electrolyte with fast ion diffusion, a high dielectric constant, and low viscosity typically delivers a low internal resistance of the battery and enables a superior rate performance and a high power density. The electrochemical potential window of an electrolyte also has a remarkably impact on the energy output by allowing for a high cell potential. In addition, high corrosivity and solvent activity of electrolytes usually cause piecemeal loss of active mass and the associated capacity fading [24, 41]. Currently, a variety of electrolytes have been developed for high-performance proton batteries, which can be categorized into liquid and solid-state electrolytes based on their physical properties (Fig. 5b). Among them, liquid electrolytes are divided into aqueous and non-aqueous electrolyte. Aqueous electrolytes can be further classified into pure phase electrolytes (Lewis acids) and hybrid electrolytes. Solid-state electrolytes can be classified into all-solid and quasi-solid electrolytes. Each electrolyte has its own advantages and disadvantage for proton battery applications.

### Pure Phase Aqueous Electrolytes

**Fig. 6:**
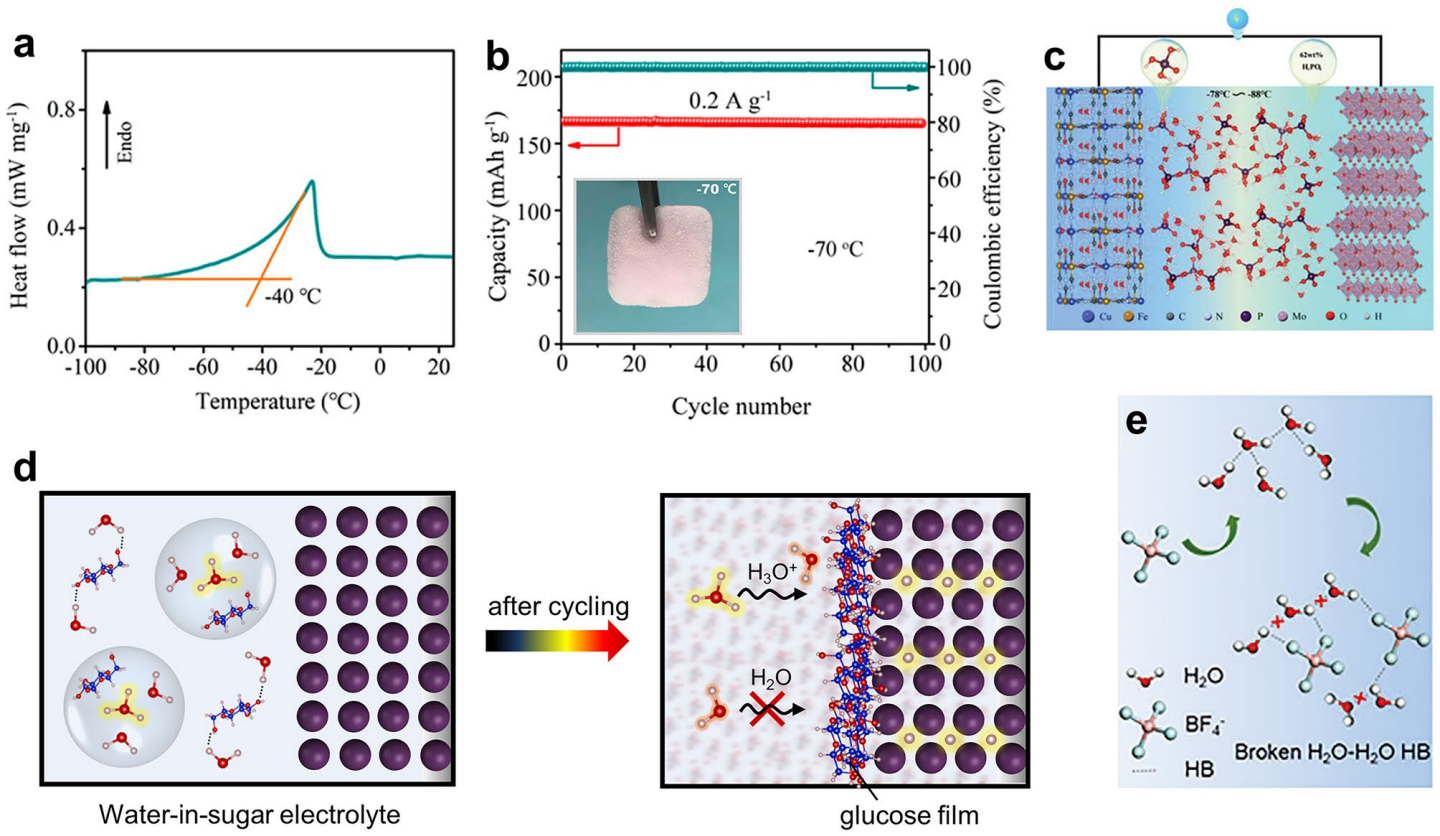
**a** Differential scanning calorimetry measurement for the 2 M H_2_SO_4_ + 2 M MnSO_4_ electrolyte. **b** Cycle performance of the MnO_2_@graphite felt//MoO_3_ cell at -70 °C. Inset: the photograph of the frozen acid electrolyte at −70 °C [17]. Copyright 2020, American Chemical Society. **c** Schematic of the aqueous proton battery device in H_3_PO_4_ electrolyte [22]. Copyright 2020, Wiley–VCH. **d** Schematic illustration of water-in-sugar electrolyte for *α*-MoO_3_ proton-battery anodes [43]. Copyright 2021, Wiley–VCH. **e** Schematic illustration on a possible mechanism of H-bond networks destruction of original water molecules by introduce BF_4_^−^ anion [75]. Copyright 2021, Wiley–VCH

Aqueous sulfuric acid electrolytes have been commonly used for proton batteries due to their good ionic conductivity (e.g., 1235 mS cm^−1^ for 4.2 M H_2_SO_4_ [43]) and a large electrochemical stability window when compared with other acids such as acetic acid, phosphoric acid and hydrochloric acid [28]. In addition, the strong hydrogen bond between SO_4_^2−^/HSO_4_^−^ and water molecules disrupts the hydrogen bond within water molecules, which could prevent ice crystallization at subzero temperatures. Thus, sulfuric acid has a low freezing point (e.g., −74 °C for 4.2 M H_2_SO_4_) with decent ion conductivity [37]. At the ultra-low temperature, proton batteries with sulfuric acid electrolytes exhibited promising electrochemical performance [64]. In the 0.5 M H_2_SO_4_ electrolyte, a Prussian blue analogues//WO_3_·nH_2_O asymmetric proton pseudocapacitor not only exhibits extraordinary rate capacities and cycling stability for proton storage at room temperature, but also delivers 70% of the room temperature capacitance and long cycle life (99% capacitance retention over 5000 cycles) at −20 °C (solid-phase electrolyte) [65, 66]. The MnO_2_@graphite felt//PTO proton full battery [19] operates well from −40 to −70 °C in frozen electrolytes of 2 M H_2_SO_4_. Even at −70 °C, the MnO_2_@graphite felt//MoO_3_ cell retains 81.5% of capacity at room temperature and an unprecedented cycle stability (~ 100% capacity maintained over 100 cycles), which mainly attributed to high ionic conductivity of the acid electrolytes (Fig. 6a, b) [17]. In addition, an aqueous Pb-quinone battery delivers impressive electrochemical performance at −70 °C (5 M H_2_SO_4_ electrolyte; liquid state), with a high discharge capacity of 87 mAh g^−1^ at 0.1 A g^−1^ and long cycle stability (97% capacity retention after 500 cycles at 0.5 A g^−1^) [67].

Nevertheless, water activities with electrode material surface in acids (mentioned in the interphase part) often causes structural distortions to electrode surface lattices, and strong aqueous acids usually corrode current collectors leading to associated capacity fading [24]. To decrease water activity and reduce the corrosivity of strong acids, the concentrated phosphoric acid (62 wt% or 9.5 M H_3_PO_4_) has been investigated by Jiang and coworkers [22], to enhance the stability of* α*-MoO_3_ anode and interfacial kinetics. The 9.5 M H_3_PO_4_ electrolyte exhibits faster proton conduction at the electrode–electrolyte interface than the dilute acid, owing to the incomplete solvation shells of protons and direct diffuse of proton from O in H_3_PO_4_ to *α*-MoO_3_ at the interphase [22]. In addition, the H_3_PO_4_ electrolyte can improve the performance of aqueous proton batteries at low temperatures [22]. The eutectic mixture 62 wt% H_3_PO_4_ delivers a low melting point of -85 °C according to the phase diagram of the mixture of H_3_PO_4_ and water, which behaves as a single liquid phase until solidification at this temperature. Thus, at −78 °C, such 9.5 M H_3_PO_4_ electrolyte not only facilitates the *α*-MoO_3_ anode-based full cell with stable cycle life and appreciable power performance, but also enables pouch cells with no capacity fading (Fig. 6c). A solvent-free protic liquid electrolyte (polyphosphoric acid) has been reported by Liao and coworkers, which combines the merits of liquid and solid electrolytes (such as nonflammability, wide electrochemical stability window, low volatility, and wide working temperature range (> 400 °C)) and enables proton full cell devices to operate well in a ultrawide temperature range of 0–250 °C [68]. Very recently, Gavriel and coworkers [50] attempted to use acetic acid as a safe and less corrosive electrolyte, which delivers substantially higher capacity with Ni[Fe(CN)_6_]_2/3_·4H_2_O cathodes than in sulfuric acids. The electrochemical quartz crystal microbalance with dissipation (EQCM-D) reveals the co-insertion of hydronium ions and protons in the host materials which contribute to more charge storage. Despite above advantages, phosphoric acid and acetic acid suffers from poor ionic conductivity (~ 60 mS cm^−1^ for 9.5 M H_3_PO_4_ [37]; ~ 2 mS cm^−1^ for 4.5 M CH_3_COOH).

Mild electrolytes such as 1 M Mg(NO_3_)_2_ [61] and ZnSO_4_ aqueous solutions [69] with intrinsic safety, low cost and environmentally friendliness also have been proposed. However, metal ions in mild electrolytes, rather than protons, are typically found to (de)intercalate from/into host materials concomitantly by the control of thermodynamics and kinetics. The materials suitable for the proton storage in the matching mild electrolyte are only limited to alloxazine and the poly(2,9-dihydroquinoxalino[2,3-b]phenazine). Another challenge for mild electrolytes is the inherent low ionic conductivity (e.g., ~ 100 mS cm^−1^ for the 1 M Mg(NO_3_)_2_ electrolyte [61]).

### Hybrid Aqueous Electrolytes

In commonly used dilute acid electrolytes, the co-interaction of water molecules and proton with electrode materials often causes electrode structural distortions for metal oxide electrodes. The adsorption of hydronium ions on electrode surfaces also facilitates HER as an unwanted side reaction. In this regard, our group [43] recently developed a “water-in-sugar” electrolyte with high concentration of sugar dissolved in aqueous acids, which stabilizes the electrode structure and delivers an extended working potential window of 3.9 V. As revealed by MD simulations, the glucose-free solutions deliver a conventional H_3_O^+^ solvation structure with three water molecules inside and sufficient free water outside. Spectroscopies and MD simulations for “water-in-sugar” electrolytes show a drastic change in H_3_O^+^ solvation sheath with glucose entering the H_3_O^+^ solvation sheath and decreased water molecules inside (Fig. 6d). Free water is also significantly decreased in electrolyte systems due to the “lock-up” of water molecules by glucose via hydrogen bonding. Taken together with the glucose-derived interphase, the water interactions with the electrode surface can be significantly suppressed with significantly improved cycling stability over 100,000 times. The significant extended potential window mostly occurs in the cathodic regions and suggests the inhibition of HER. In addition, the ultrafast and diffusion-free Grotthuss proton conduction in the “water-in-sugar” electrolyte also provides a high ionic conductivity (258.4 mS cm^−1^) for achieving an outstanding rate performance from 4 to 40 A g^−1^.

Although “water-in-sugar” electrolyte delivers reduced water activity and enhanced electrode stability, this electrolyte is not appropriate for low-temperature operations as sugar becomes less soluble with the decreasing temperature. Very recently, our group demonstrates a water–water hydrogen bond disrupting electrolyte using the non-toxic and low-cost cryoprotectants (such as glycerol, methanol, ethanol, acetic acid, ethylene glycol) mixed with acids (such as H_2_SO_4_, H_3_PO_4_ and HCl) for proton batteries [37]. Hydrogen bonds involving water molecules are disrupted by the introduction of cryoprotectants, leading to a very low freezing point (less than −100 °C), minimized water activity, and also a modified hydronium ion solvation sheaths. Concomitantly, glycerol binds preferentially to the electrode surface to protect it from water attacking. This electrolyte delivers a decent ion conductivity (126.9 mS cm^–1^). Fast and stable proton storage is achieved in the K_0.2_VO_0.6_[Fe(CN)_6_]_0.8_·4.1H_2_O//MoO_3_ full cell, even at temperatures as low as −50 °C. Very similar as aqueous Zn-ion batteries, organic electrolyte additives in proton batteries can simultaneously regulate solvation shell and electrode interface, significantly suppress the water-induced corrosion reactions and HER, and thereby enhance the electrochemical stability of anodes [70]. Besides hindering the severe by-reactions derived from active water, organic additives in Zn-ion batteries (e.g., polyethylene oxide, polyacrylamide, glucose, and methanol [71–74]) can adsorb on the Zn foil anode, influence the formation of initial nuclei, and then inhibit the following Zn dendrite growth.

Apart from the acid-organic hybrid electrolytes, there are also acid–salt and acid–acid hybrid aqueous electrolyte systems as well as protic ionic liquid hybrid electrolytes. To achieve high performance at low temperatures, 2 M HBF_4_ + 2 M Mn(BF_4_)_2_ electrolyte [75] has been developed with a ultralow freezing point below −160 °C, due to the efficient break of hydrogen-bond networks of water molecules via the introduction of BF_4_^−^ anions (Fig. 6e). The proton battery with alloxazine anode and MnO_2_/Mn^2+^ conversion in carbon felt cathode can operate even at −90 °C and still show a high specific energy density of 110 Wh kg^−1^ at a specific power density of 1,650 W kg^−1^ at −60 °C. A novel and low-cost “water in salt” electrolyte via dissolving 20 M ZnCl_2_ in 1 M HCl has also been developed by Yang et al. [76], which enables a stable MoO_3_//K_2_NiFe(CN)_6_·1.05H_2_O (Ni-PBA) full cell. The acid-acid hybrid electrolytes (5 M H_2_SO_4_ + 3 M H_3_PO_4_) have also developed, where the enhanced hydrogen bonds in electrolytes significantly reduces the dissolution of anode materials [77]. In addition, protic ionic liquids have been employed by Sjödin et al. [78] as the electrolytes for all-organic proton batteries. In the 0.1 M MeTriHTFSI/acetonitrile (MeCN)/H_2_O electrolyte, a high potential (0.8 V) proton rocking-chair battery is achieved with the conducting redox polymer electrodes, quinizarin and naphthoquinone.

### Non-Aqueous Electrolytes

**Fig. 7:**
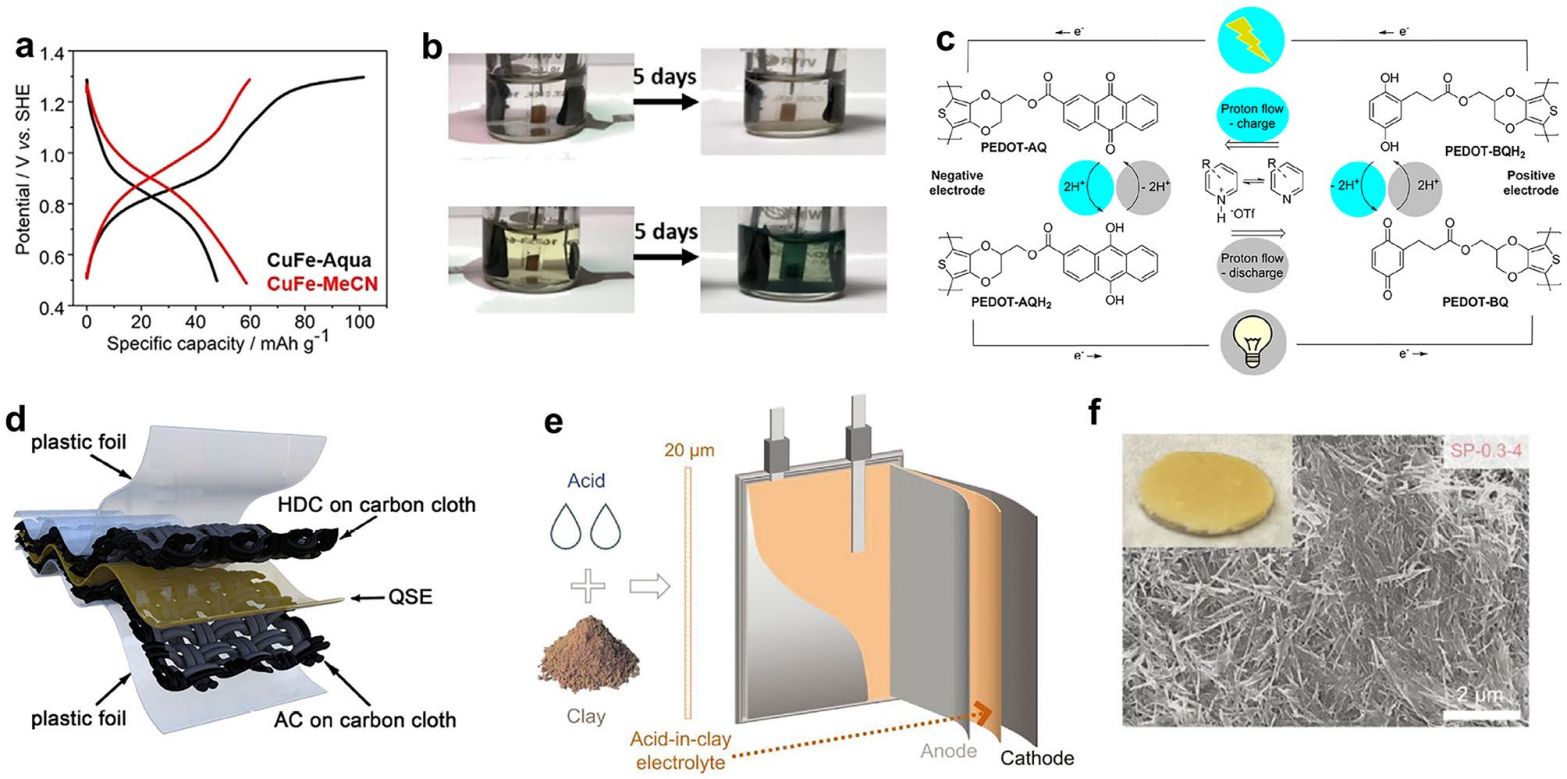
**a** Galvanostatic charge/discharge profiles of the first cycle of Cu[Fe(CN)_6_]_0.63_·□_0.37_·3.4H_2_O in aqueous and non-aqueous electrolytes. **b** Digital images of beaker cells of Mn[Fe(CN)_6_]_0.63_·□_0.37_·3.4H_2_O before and after cycling at 10 mAg^−1^ for five days in aqueous and non-aqueous electrolytes [41]. Copyright 2020, Wiley–VCH. **c** The schematic illustration of the all-organic poly(3,4-ethylenedioxythiophene)-benzonquinone (PEDOT-BQ)//poly(3,4-ethylenedioxythiophene)-anthraquinone proton battery [79]. Copyright 2017, American Chemical Society. **d** Schematic diagram of the metal-free quasi-solid flexible soft-packed battery [60]. Copyright 2022, Wiley–VCH. **e** Schematic diagram and **f** SEM image of acid-in-clay electrolytes. Inset of **f**: photograph by a camera [80]. Copyright 2022, Wiley–VCH

Besides aqueous electrolytes, non-aqueous proton electrolytes have also been devised. Organic electrolytes can not only tackle the challenge of electrode material dissolution with less solvent-electrode interactions, but also deliver a much wider electrochemical window allowing for a high-voltage proton full cell. To avoid the high corrosivity of strong acids, Xu and coworkers prepared the 1 M H_3_PO_4_ in MeCN [41]. Such an electrolyte shows unique characteristics compared to conventional aqueous acidic electrolytes (H_3_PO_4_/H_2_O): (*i*) higher (de)protonation potential (Fig. 7a) and a lower desolvation energy of protons in the electrode–electrolyte interface; (*ii*) better cycling stability by dissolution suppression (Fig. 7b); (*iii*) higher Coulombic efficiency (CE) owing to the suppression of oxygen evolution reaction. With this non-aqueous electrolyte, the proton full cells based on Prussian blue analogues cathodes and *α*-MoO_3_ anodes deliver stable cycling performance and less self-discharge compared to the aqueous counterpart. Another organic electrolyte example comes from the all-organic proton battery proposed by Sjödin et al. (Fig. 7c) [79]. The electrolyte used is an ionic liquid-type slurry, a MeCN solution of organic acids and bases (substituted pyridinium triflates and the corresponding pyridine base). This slurry allows the quinone/hydroquinone redox reaction of organic electrodes and suppresses the proton reduction in the meantime. Nevertheless, non-aqueous electrolytes generally suffer from low ionic conductivity (e.g., only 0.5 mS cm^−1^ for H_3_PO_4_/MeCN electrolyte), and are typically flammable and volatile, bring safety issues for battery systems.

### Solid/Quasi-Solid Electrolytes

To overcome the issue of corrosion, broaden the electrolyte potential window and inhibit the solvent-electrode interactions, solid and quasi-solid electrolytes have attracted recent research interest. A novel family of solid proton electrolytes (acid-in-clay electrolyte) have been reported recently by Wang et al. [80] via integrating proton charge carriers in a natural phyllosilicate clay network that can be manufactured into thin-film (tens of microns) fluid-impervious membranes (Fig. 7e, f). The chosen example systems (sepiolite-phosphoric acid) rank first among the solid proton conductors in terms of reduced chemical activity, electrochemical stability window (3.35 V), and proton conductivities (15 mS cm^−1^ at 25 °C, 0.023 mS cm^−1^ at −82 °C). Benefitting from the advantages of superfast proton transport, the wide electrochemical stability window, reduced corrosivity, and excellent ionic selectivity of acid-in-clay electrolytes, the two main challenges of proton batteries (gassing and poor cyclability) have been successfully solved. Solid full batteries can cycle 20,000 times at 1000 mA g^−1^ with only 26% capacity decay at room temperature and deliver no capacity decay after 3000 cycles at 100 mA g^−1^ at −20 °C. In addition, the frozen acids can be regarded as a special kind of solid-state electrolyte. The 2 M H_2_SO_4_ + 2 M MnSO_4_ electrolyte was completely frozen to ice-like solids at −70 °C (Fig. 7b) and facilitated the operation of solid-state proton batteries (e.g., MnO_2_@graphite felt//PTO and MnO_2_@graphite felt//MoO_3_ full cells) [17, 19]. Despite less corrosivity and enhanced cycling stability, frozen acids can only operate at freezing temperatures.

Shen and coworkers [60] have developed a quasi-solid electrolyte using 1 M H_2_SO_4_ aqueous solution and polyvinyl alcohol, as shown in Fig. 7d. With this as-prepared electrolyte, the 2,5-dichloro-1,4-phenylene bis((ethylsulfonyl)amide) cathode retains 89.67% of original capacity after 2000 cycles. A flexible metal-free quasi-solid battery is also developed showing an excellent electrochemical performance. Using a flexible and binder-free redox-active polymer@MXene electrode, Shi et al. [81] have developed a flexible proton battery device based on the polyvinyl alcohol-H_2_SO_4_ gel electrolyte, which delivers a considerable energy density, a supercapacitor-level power density, and a record lifespan.

### Electrolyte Analysis Techniques

Based on Fig. 5a and recent progress in proton battery electrolytes, we summarize four categories of analysis techniques for the study of electrolyte physicochemical properties, water activity, solvation structure, and potential window. (*i*) The common physicochemical properties for proton batteries electrolytes include ionic conductivity, viscosity, and freezing point (and/or boiling point), which can be investigated by the conductivity meter (or EIS technology [37]), kinematic viscometer, and differential scanning calorimetry (DSC), respectively. For example, the ionic conductivities of hydrogen-bond disrupting electrolytes were investigated by EIS with two parallel Ti-plate electrodes (1 cm × 1 cm) as electrodes [37]. (*ii*) The free water fraction in aqueous electrolytes can be calculated by MD simulations. The water activity in electrolytes can also be reflected by spectroscopies, such as Raman, Fourier transform infrared spectroscopy (FTIR), and liquid NMR. (*iii*) The solvation structure of electrolytes is difficult to be uncovered by experimental techniques but can be revealed by MD simulations. (*iv*) The potential window of electrolytes can be determined via CV tests by a three-electrode cell with platinum, gold and/or glassy carbon electrodes as working electrodes. Su et al. reported a 3.9 V working potential window for “water-in-sugar” electrolytes via a glassy carbon working electrode [43].

## Conclusion and Perspective

The recent progress on the development of electrode–electrolyte interphase and electrolytes for aqueous proton battery technologies have been summarized. Despite its importance, current understanding of electrode–electrolyte interphase is limited. Based on the type of insertion ions (protons or hydronium ions) into bulk lattices, we classify the desolvation process of proton-storage materials into two categories, complete desolvation and incomplete desolvation. The complete desolvation of hydrated protons (namely naked proton insertion) happens in *α*-MoO_3_ and anatase TiO_2_, while the incomplete dehydration (hydronium ion insertion) occurs in hexagonal WO_3_·0.6H_2_O and most organic solids. Water activities at the interphase strongly affect the structure stability of surface lattice and the whole battery lifespan. The interfacial reaction kinetics also play a critical role on the rate performance. Artificial electrode interphases fabricated by electrode design (TiO_2_ coating) and electrolyte engineering (glucose-derived film) have been reviewed to improve the cycling stability and rate capability of proton batteries.

To further advance the field of high-performance proton batteries and understand the interphase reaction mechanism, several important areas require further investigations, which are listed as follows:

i. Discoveries of more proton-storage materials, such as the PBAs, MXenes, and organic solids, are highly required. Interfacial reaction kinetics, solvent-electrode surface interactions, desolvation process, and the type of inserted charge carriers in these materials should be understood. Advanced new analytical techniques, such as in situ optical microscopy, cryogenic electron microscopy and in situ transmission electron microscopy can be applied to investigate the charge transfer reactions, reaction mechanism, microstructure, morphology, and gas evolutions.
ii. More strategies on electrode design and electrolyte engineering can be considered for proton batteries to improve their stability and reaction kinetics. The introduction of additional surface layers (e.g., metal oxides, carbon materials, organic compounds) can not only promote the desolvation of hydrated protons, but also protect the electrode surface from water attacking. The addition of organic agents, polymers, co-solvents, and/or salts into the electrolytes is an effective strategy to in situ construct interphase layers. Tailoring the solvation structure of electrolytes to adjust the reduction potential is a promising approach to achieve the robust film on electrode surface [5, 82]. In addition to the in-depth mechanistic understanding on the interphase formation mechanism and interphase reaction kinetics, investigations in the future should also focus on the long-term stability of interphase layer and self-discharge rates.
iii. Design of materials for direct insertion of hydrated protons (i.e., hydronium ions) to facilitate the desolvation process and interface reaction kinetics. As hydronium ion (H_3_O^+^) has a larger size than proton, the co-insertion of hydronium ions and protons generally requires electrode materials to have layered structures and/or open frameworks with large ionic channels. Organic solids with large planar structure and the metal ions or water molecules engaged metal oxides with enlarged layer spacings can be developed to enable fast proton storage.

**Fig. 8:**
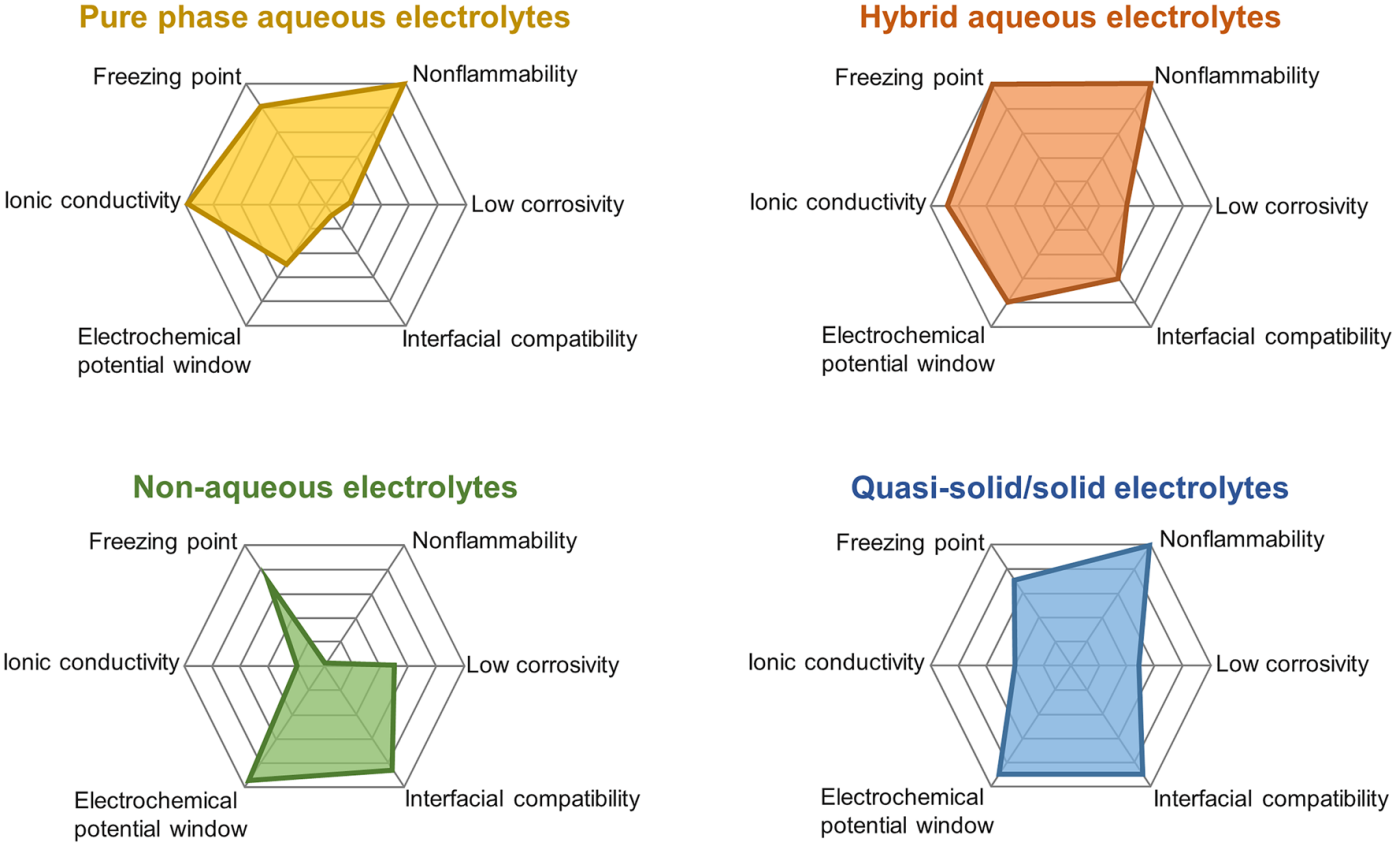
Comparison of electrolytes for proton batteries in aspects of their different physical and electrochemical properties

Electrolytes is of paramount importance for proton batteries and can directly determine the energy density, power density, cycle life, safety, and operating conditions of proton batteries. According to their physical properties, we have categorized the current developed electrolytes into liquid electrolytes (pure phase aqueous electrolytes, hybrid aqueous electrolytes, and non-aqueous electrolytes) and solid/quasi-solid electrolytes. Figure 8 summarizes the physical and electrochemical properties of the four types of electrolytes. Although pure acids have been regarded as default electrolytes for most proton batteries, they exhibit a low energy density, high water activities, and strong corrosivity. The strong water–electrode interactions also cause the fast dissolution of electrode materials and inferior cycle life. To decrease the water activity and the freezing point and extend the potential window, organic agents, salt, and acids are subsequently added to original acid electrolytes to generate hybrid electrolytes. Non-aqueous electrolytes can also deliver a enlarged electrochemical potential window, improved electrode stability, and low corrosivity, but they suffer from issues like flammability and very low ionic conductivity. It is anticipated that solid/quasi-solid electrolytes exhibiting nonflammability, wide electrochemical stability window, decent ionic conductivity, reduced corrosivity, and reduced chemical reactivity can significantly facilitate the development of proton battery towards a new-generation portable energy device.

Several challenges remain in the development of suitable high-performance electrolytes for application in proton batteries. Some perspectives on the study of electrolytes are proposed as follows:

i. The accessible electrochemical stability window for aqueous electrolytes needs further improvements. Resembling the water-in-salt electrolyte (21 M lithium bis(trifluoromethanesulfonyl) imide), concentrated electrolytes can be adopted to proton batteries to extend the operation voltage window, which will offer more choices of electrode materials in the expanded voltage window. However, a balance is required between energy density and cost. In this regard, the concentrated electrolytes with pricey organic molecules and/or salts should be revaluated.
ii. New strategies are required to decrease the water activity in commonly used aqueous electrolytes. Various strategies including concentrated acids, frozen acids, non-aqueous electrolytes, and concentrated electrolytes have been developed, but all suffer from low ionic conductivity. Therefore, finding an optimal balance between water activity and ionic conductivity is one of the key tasks. A variety of weak acids, buffer solutions, ionic liquids, or proton mediators (such as the electrolyte of protonated pyridinium triflate derivate in acetonitrile used by Sjödin and coworkers [79]) with moderate conductivity can be explored as proton-storage electrolytes.
iii. Solid proton batteries are promising because protons can transport rapidly via the special Grotthuss conduction in solid electrolytes, compared with metal-ion solid batteries with inferior ionic conductivity. Currently, there is only one report about the acid-in-clay film [80]. More investigations on solid proton electrolytes are highly needed. Solid electrolytes will enable proton batteries to be used in flexible and portable energy storage devices for many applications in daily life. For actual operation in the future, stacking all-solid-state proton batteries will be essential to expand cell voltages and increase the energy density; however, stack structure (from the example of the fuel cells) usually generates a large amount of Joule heat. In this regard, solid-state electrolytes and electrode materials with high heat resistance at 100 °C or higher should be rationally developed. For example, a proton-conducting Sn_0.95_Al_0.05_H_0.05_P_2_O_7_-polytetrafluoroethylene composite electrolyte has been developed for high-temperature supercapacitors, and porous carbon modified with carbonyl groups has been designed as rechargeable proton exchange membrane (PEM) fuel-cell batteries anode materials which can operate at high temperature [83–86]. In terms of screening electrode materials and electrolytes for future proton batteries, artificial intelligence (AI; e.g., machine learning, ML) is a promising technique. Although the data pool for aqueous proton batteries is relatively limited for now, predictions is applicable via referring to ML models of other aqueous batteries systems, such as Li^+^, Na^+^, K^+^ and Zn^2+^. Further, the amount of proton battery data (or publications) may grow exponentially like LIBs, where it may be difficult for researchers to read through all data; hence, artificial intelligence with the strong data processing capability could be very necessary.

## References

[1] Yang Z, Zhang J, Kintner-Meyer MCW, Lu X, Choi D (2011). Electrochemical energy storage for green grid. Chem. Rev..

[2] Reddy MV, Subba Rao GV, Chowdari BVR (2013). Metal oxides and oxysalts as anode materials for Li ion batteries. Chem. Rev..

[3] Zhu GN, Wang YG, Xia YY (2012). Ti-based compounds as anode materials for Li-ion batteries. Energy Environ. Sci..

[4] Hu X, Zhang W, Liu X, Mei Y, Huang Y (2015). Nanostructured Mo-based electrode materials for electrochemical energy storage. Chem. Soc. Rev..

[5] Suo L, Borodin O, Gao T, Olguin M, Ho J (2015). “Water-in-salt” electrolyte enables high-voltage aqueous lithium-ion chemistries. Science.

[6] Wang H, Tan R, Yang Z, Feng Y, Duan X (2021). Stabilization perspective on metal anodes for aqueous batteries. Adv. Energy Mater..

[7] Kim DJ, Yoo DJ, Otley MT, Prokofjevs A, Pezzato C (2019). Rechargeable aluminium organic batteries. Nat. Energy.

[8] Nam KW, Kim H, Beldjoudi Y, Kwon TW, Kim DJ (2020). Redox-active phenanthrenequinone triangles in aqueous rechargeable zinc batteries. J. Am. Chem. Soc..

[9] Su D, McDonagh A, Qiao SZ, Wang G (2017). High-capacity aqueous potassium-ion batteries for large-scale energy storage. Adv. Mater..

[10] Gao H, Goodenough JB (2016). An aqueous symmetric sodium-ion battery with NASICON-structured Na_3_MnTi(PO_4_)_3_. Angew. Chem. Int. Ed..

[11] Wang F, Borodin O, Gao T, Fan X, Sun W (2018). Highly reversible zinc metal anode for aqueous batteries. Nat. Mater..

[12] Xu Y, Wu X, Ji X (2021). The renaissance of proton batteries. Small Struct..

[13] Yu H, So Y, Kuwabara A, Tochigi E, Shibata N (2016). Crystalline grain interior confi guration affects lithium migration kinetics in Li-rich layered oxide. Nano Lett..

[14] Yasuda T, Watanabe M (2013). Protic ionic liquids: fuel cell applications. MRS Bull..

[15] Dong S, Shin W, Jiang H, Wu X, Li Z (2019). Ultra-fast NH^4+^ storage: strong H bonding between NH^4+^ and Bi-layered V_2_O_5_. Chem.

[16] Xu T, Wang D, Li Z, Chen Z, Zhang J (2022). Electrochemical proton storage: from fundamental understanding to materials to devices. Nano-Micro Lett..

[17] L. Yan, J. Huang, Z. Guo, X. Dong, Z. Wang et al., Solid-state proton battery operated at ultralow temperature. ACS Energy Lett. 685–691 (2020). 10.1021/acsenergylett.0c00109

[18] Wang X, Bommier C, Jian Z, Li Z, Chandrabose RS (2017). Hydronium-ion batteries with perylenetetracarboxylic dianhydride crystals as an electrode. Angew. Chem. Int. Ed..

[19] Guo Z, Huang J, Dong X, Xia Y, Yan L (2020). An organic/inorganic electrode-based hydronium-ion battery. Nat. Commun..

[20] Yu J, Li J, Leong ZY, Sheng Li D, Lu J (2021). A crystalline dihydroxyanthraquinone anodic material for proton batteries. Mater. Today Energy.

[21] Su Z, Ren W, Guo H, Peng X, Chen X (2020). Ultrahigh areal capacity hydrogen-ion batteries with MoO_3_ loading over 90 mg cm^−2^. Adv. Funct. Mater..

[22] Jiang H, Shin W, Ma L, Hong JJ, Wei Z (2020). A high-rate aqueous proton battery delivering power below −78 °C via an unfrozen phosphoric acid. Adv. Energy Mater..

[23] Wang X, Xie Y, Tang K, Wang C, Yan C (2018). Redox chemistry of molybdenum trioxide for ultrafast hydrogen-ion storage. Angew. Chem. Int. Ed..

[24] Guo H, Goonetilleke D, Sharma N, Ren W, Su Z (2020). Two-phase electrochemical proton transport and storage in α-MoO_3_ for proton batteries. Cell Rep. Phys. Sci..

[25] Jiang H, Hong JJ, Wu X, Surta TW, Qi Y (2018). Insights on the proton insertion mechanism in the electrode of hexagonal tungsten oxide hydrate. J. Am. Chem. Soc..

[26] Geng C, Sun T, Wang Z, Wu JM, Gu YJ (2021). Surface-induced desolvation of hydronium ion enables anatase TiO_2_ as an efficient anode for proton batteries. Nano Lett..

[27] Wang S, Zhao X, Yan X, Xiao Z, Liu C (2019). Regulating fast anionic redox for high-voltage aqueous hydrogen-ion-based energy storage. Angew. Chem. Int. Ed..

[28] Wu X, Hong JJ, Shin W, Ma L, Liu T (2019). Diffusion-free Grotthuss topochemistry for high-rate and long-life proton batteries. Nat. Energy.

[29] Peng X, Guo H, Ren W, Su Z, Zhao C (2020). Vanadium hexacyanoferrate as high-capacity cathode for fast proton storage. Chem. Commun..

[30] Li W, Xu C, Yang Z, Yu H, Li W (2022). Sodium manganese hexacyanoferrate as ultra-high rate host for aqueous proton storage. Electrochim. Acta.

[31] Jiang H, Shin W, Ma L, Hong JJ, Wei Z (2020). A high-rate aqueous proton battery delivering power below −78 °C via an unfrozen phosphoric acid. Adv. Energy Mater..

[32] Wang F, Suo L, Liang Y, Yang C, Han F (2017). Spinel LiNi_0.5_Mn_1.5_O_4_ cathode for high-energy aqueous lithium-ion batteries. Adv. Energy Mater..

[33] Shen Y, Han X, Cai T, Hu H, Li Y (2020). High-performance aqueous sodium-ion battery using a hybrid electrolyte with a wide electrochemical stability window. RSC Adv..

[34] Li J, Yan H, Xu C, Liu Y, Zhang X (2021). Insights into host materials for aqueous proton batteries: structure, mechanism and prospect. Nano Energy.

[35] Xu C, Yang Z, Zhang X, Xia M, Yan H (2021). Prussian blue analogues in aqueous batteries and desalination batteries. Nano-Micro Lett..

[36] Liu H, Cai X, Zhi X, Di S, Zhai B (2023). An amorphous anode for proton battery. Nano-Micro Lett..

[37] Su Z, Chen J, Stansby J, Jia C, Zhao T (2022). Hydrogen-bond disrupting electrolytes for fast and stable proton batteries. Small.

[38] Zheng J, Hou Y, Duan Y, Song X, Wei Y (2015). Janus solid-liquid interface enabling ultrahigh charging and discharging rate for advanced lithium-ion batteries. Nano Lett..

[39] Gaberscek M, Dominko R, Jamnik J (2007). Is small particle size more important than carbon coating? An example study on LiFePO_4_ cathodes. Electrochem. Commun..

[40] Malik R, Abdellahi A, Ceder G (2013). A critical review of the Li insertion mechanisms in LiFePO_4_ electrodes. J. Electrochem. Soc..

[41] Xu Y, Wu X, Jiang H, Tang L, Koga KY (2020). A non-aqueous H_3_PO_4_ electrolyte enables stable cycling of proton electrodes. Angew. Chem. Int. Ed..

[42] Donald WA, Leib RD, Demireva M, Brien JTO, Prell J (2009). Directly relating reduction energies of Gaseous Eu(H_2_O)_n_^3+^, n = 55–140, to aqueous solution: the absolute SHE potential and real proton solvation energy. J. Am. Chem. Soc..

[43] Su Z, Chen J, Ren W, Guo H, Jia C (2021). “Water-in-sugar” electrolytes enable ultrafast and stable electrochemical naked proton storage. Small.

[44] Su Z, Tang J, Chen J, Guo H, Wu S (2023). Co-insertion of water with protons into organic electrodes enables high-rate and high-capacity proton batteries. Small Struct..

[45] Lu Y, Wang L, Cheng J, Goodenough JB (2012). Prussian blue: a new framework of electrode materials for sodium batteries. Chem. Commun..

[46] Lee JS, Nam G, Sun J, Higashi S, Lee HW (2016). Composites of a prussian blue analogue and gelatin-derived nitrogen-doped carbon-supported porous spinel oxides as electrocatalysts for a Zn–air battery. Adv. Energy Mater..

[47] Wu X, Xu Y, Jiang H, Wei Z, Hong JJ (2018). NH^4+^ topotactic insertion in berlin green: an exceptionally long-cycling cathode in aqueous ammonium-ion batteries. ACS Appl. Energy Mater..

[48] Qiao J, Qin M, Shen YM, Cao J, Chen Z (2021). A rechargeable aqueous proton battery based on a dipyridophenazine anode and an indium hexacyanoferrate cathode. Chem. Commun..

[49] Wu X, Qiu S, Xu Y, Ma L, Bi X (2020). Hydrous nickel-iron turnbull’s blue as a high-rate and low-temperature proton electrode. ACS Appl. Mater. Interfaces.

[50] Gavriel B, Bergman G, Turgeman M, Nimkar A, Elias Y (2023). Aqueous proton batteries based on acetic acid solutions: mechanistic insights. Mater. Today Energy.

[51] Xu K (2019). Diffusionless charge transfer. Nat. Energy.

[52] Luo W, Allen M, Raju V, Ji X (2014). An organic pigment as a high-performance cathode for sodium-ion batteries. Adv. Energy Mater..

[53] Rodríguez-Pérez IA, Jian Z, Waldenmaier PK, Palmisano JW, Chandrabose RS (2016). A hydrocarbon cathode for dual-ion batteries. ACS Energy Lett..

[54] Han X, Chang C, Yuan L, Sun T, Sun J (2007). Aromatic carbonyl derivative polymers as high-performance Li-ion storage materials. Adv. Mater..

[55] Liang Y, Chen Z, Jing Y, Rong Y, Facchetti A (2015). Heavily n-dopable π-conjugated redox polymers with ultrafast energy storage capability. J. Am. Chem. Soc..

[56] Muench S, Wild A, Friebe C, Häupler B, Janoschka T (2016). Polymer-based organic batteries. Chem. Rev..

[57] Liang Y, Tao Z, Chen J (2012). Organic electrode materials for rechargeable lithium batteries. Adv. Energy Mater..

[58] Lin K, Chen Q, Gerhardt MR, Tong L, Kim SB (2015). Alkaline quinone flow battery. Science.

[59] Liang Y, Jing Y, Gheytani S, Lee KY, Liu P (2017). Universal quinone electrodes for long cycle life aqueous rechargeable batteries. Nat. Mater..

[60] Shen D, Rao AM, Zhou J, Lu B (2022). High-potential cathodes with nitrogen active centres for quasi-solid proton-ion batteries. Angew. Chem. Int. Ed..

[61] Sun T, Liu C, Xu XF, Nian Q, Zheng S (2020). Insights into the hydronium-ion storage of alloxazine in mild electrolyte. J. Mater. Chem. A.

[62] Wang C, Zhao S, Song X, Wang N, Peng H (2022). Suppressed dissolution and enhanced desolvation in core–shell MoO_3_@TiO_2_ nanorods as a high-rate and long-life anode material for proton batteries. Adv. Energy Mater..

[63] Wang C, Zhao S, Song X, Wang N, Peng H (2022). Suppressed dissolution and enhanced desolvation in core–shell MoO_3_@TiO_2_ nanorods as a high-rate and long-life anode material for proton batteries. Adv. Energy Mater..

[64] Yue F, Tie Z, Deng S, Wang S, Yang M (2021). An ultralow temperature aqueous battery with proton chemistry. Angew. Chem. Int. Ed..

[65] Wang D, Xu T, Zhang M, Ren Z, Tong H (2023). A novel layered WO_3_ derived from an ion etching engineering for ultrafast proton storage in frozen electrolyte. Adv. Funct. Mater..

[66] Xu T, Li Z, Wang D, Zhang M, Ai L (2022). A fast proton-induced pseudocapacitive supercapacitor with high energy and power density. Adv. Funct. Mater..

[67] Yue F, Tie Z, Deng S, Wang S, Yang M (2021). An ultralow temperature aqueous battery with proton chemistry. Angew. Chem. Int. Ed..

[68] Liao M, Ji X, Cao Y, Xu J, Qiu X (2022). Solvent-free protic liquid enabling batteries operation at an ultra-wide temperature range. Nat. Commun..

[69] Tie Z, Deng S, Cao H, Yao M, Niu Z (2022). A symmetric all-organic proton battery in mild electrolyte. Angew. Chem. Int. Ed..

[70] Yuan L, Hao J, Kao CC, Wu C, Liu HK (2021). Regulation methods for the Zn/electrolyte interphase and the effectiveness evaluation in aqueous Zn-ion batteries. Energy Environ. Sci..

[71] Jin Y, Han KS, Shao Y, Sushko ML, Xiao J (2020). Stabilizing zinc anode reactions by polyethylene oxide polymer in mild aqueous electrolytes. Adv. Funct. Mater..

[72] Zhang Q, Luan J, Fu L, Wu S, Tang Y (2019). The three-dimensional dendrite-free zinc anode on a copper mesh with a zinc-oriented polyacrylamide electrolyte additive. Angew. Chem. Int. Ed..

[73] Sun P, Ma L, Zhou W, Qiu M, Wang Z (2021). Simultaneous regulation on solvation shell and electrode interface for dendrite-free Zn ion batteries achieved by a low-cost glucose additive. Angew. Chem. Int. Ed..

[74] Hao J, Yuan L, Ye C, Chao D, Davey K (2021). Boosting zinc electrode reversibility in aqueous electrolytes by using low-cost antisolvents. Angew. Chemie.

[75] Sun T, Du H, Zheng S, Shi J, Tao Z (2021). High power and energy density aqueous proton battery operated at −90 °C. Adv. Funct. Mater..

[76] Yang B, Qin T, Du Y, Zhang Y, Wang J (2022). Rocking-chair proton battery based on a low-cost “water in salt” electrolyte. Chem. Commun..

[77] Dai R, Liu H, Zhi X, Di S, Zhai B (2022). A composite acidic electrolyte for ultra-long-life hydrogen-ion storage. Chem. Eng. J..

[78] Wang H, Emanuelsson R, Karlsson C, Jannasch P, Strømme M (2021). Rocking-chair proton batteries with conducting redox polymer active materials and protic ionic liquid electrolytes. ACS Appl. Mater. Interfaces.

[79] Emanuelsson R, Sterby M, Strømme M, Sjödin M (2017). An all-organic proton battery. J. Am. Chem. Soc..

[80] Wang S, Jiang H, Dong Y, Clarkson D, Zhu H (2022). Acid-in-clay electrolyte for wide-temperature-range and long-cycle proton batteries. Adv. Mater..

[81] Shi M, Wang R, Li L, Chen N, Xiao P (2022). Redox-active polymer integrated with MXene for ultra-stable and fast aqueous proton storage. Adv. Funct. Mater..

[82] Ni Q, Kim B, Wu C, Kang K (2022). Non-electrode components for rechargeable aqueous zinc batteries: electrolytes, solid-electrolyte-interphase, current collectors, binders, and separators. Adv. Mater..

[83] Hibino T, Kobayashi K, Nagao M, Yamamoto Y (2016). Design of a rechargeable fuel-cell battery with enhanced performance and cyclability. J. Electrochem. Soc..

[84] Nagao M, Kobayashi K, Yamamoto Y, Hibino T (2015). Rechargeable PEM fuel-cell batteries using quinones as hydrogen carriers. J. Electrochem. Soc..

[85] Kobayashi K, Nagao M, Yamamoto Y, Heo P, Hibino T (2015). Rechargeable PEM fuel-cell batteries using porous carbon modified with carbonyl groups as anode materials. J. Electrochem. Soc..

[86] Hibino T, Kobayashi K, Nagao M, Kawasaki S (2015). High-temperature supercapacitor with a proton-conducting metal pyrophosphate electrolyte. Sci. Rep..

[87] Yang X, Ni Y, Lu Y, Zhang Q, Hou J (2022). Designing quinone-based anodes with rapid kinetics for rechargeable proton batteries. Angew. Chem. Int. Ed..

